# Prototype Early Warning Systems for Vector-Borne Diseases in Europe

**DOI:** 10.3390/ijerph120606333

**Published:** 2015-06-02

**Authors:** Jan C. Semenza

**Affiliations:** European Centre for Disease Prevention and Control, Tomtebodavagen 11A, SE-171 83 Stockholm, Sweden; E-Mail: Jan.Semenza@ecdc.europa.eu; Tel.: +46-8-5860-1217; Fax: +46-8-5860-1296

**Keywords:** vector-borne diseases, early warning, climate change, globalization, malaria, West Nile fever, dengue.

## Abstract

Globalization and environmental change, social and demographic determinants and health system capacity are significant drivers of infectious diseases which can also act as epidemic precursors. Thus, monitoring changes in these drivers can help anticipate, or even forecast, an upsurge of infectious diseases. The European Environment and Epidemiology (E3) Network has been built for this purpose and applied to three early warning case studies: (1) The environmental suitability of malaria transmission in Greece was mapped in order to target epidemiological and entomological surveillance and vector control activities. Malaria transmission in these areas was interrupted in 2013 through such integrated preparedness and response activities. (2) Since 2010, recurrent West Nile fever outbreaks have ensued in South/eastern Europe. Temperature deviations from a thirty year average proved to be associated with the 2010 outbreak. Drivers of subsequent outbreaks were computed through multivariate logistic regression models and included monthly temperature anomalies for July and a normalized water index. (3) Dengue is a tropical disease but sustained transmission has recently emerged in Madeira. Autochthonous transmission has also occurred repeatedly in France and in Croatia mainly due to travel importation. The risk of dengue importation into Europe in 2010 was computed with the volume of international travelers from dengue affected areas worldwide.These prototype early warning systems indicate that monitoring drivers of infectious diseases can help predict vector-borne disease threats.

## 1. Introduction 

The emergence and spread of vector-borne diseases (VBD) in Europe is a function of biotic (living organisms in an ecosystem), abiotic (non-living elements in an ecosystem) and socioeconomic drivers of disease. Permissive circumstances that coincide in time and space can trigger an outbreak of VBD. Anticipating and elucidating such an outbreak requires a systems perspective. One such approach would be to conduct a foresight study in order to identify and monitor new developments or changes regarding a specific issue, or to develop new tools, such as scenarios, to inform strategic planning, research prioritizations, policy-making, *etc.* [1]. In such a foresight study, the European Centre for Disease Prevention and Control (ECDC) mapped the interrelated and interdependent nature of disease drivers, in order to predict the abrupt emergence of infectious disease threats by 2020 in Europe [2]. The most significant infectious disease drivers for Europe were grouped into three broad categories: globalization and environmental change; social and demographic change; and public health system. Their relation to VBD is briefly described below:

(1) Globalization and environmental change are recognized as significant disease drivers. They include the steadily expanding reach of travel and trade and population movements. Global disease dispersal is aided by a dense network of air traffic and shipping routes [3,4]. They have facilitated the arrival, establishment and spread of invasive pathogens to novel geographic destinations, including dengue virus, malaria, chikungunya virus, and West Nile virus [5]. Approximately 60% of human pathogens are estimated to be of zoonotic origin, in that they can be transmitted from animals to humans [6]. Thus, land-use can indirectly determine the spread of zoonotic diseases through different exposure pathways in urban, sub-urban and rural settings with a range of animal habitats such as pastures, arable fields, and managed forests [6,7]. Urbanization, urban sprawl and high population densities have also been associated with VBD emergence [8]. Habitat encroachment and habitat destruction can result in displacement of wild animals into novel environments which can have a bearing on exposure patterns to infectious pathogens. Climatic conditions are also significant drivers of VBD since some of the vectors are cold-blooded; thus, climate change can shift the geographical ranges of VBD transmission [9,10,11,12,13,14].

(2) Social and demographic change includes the shift in demographic profile, social inequality and lifestyle. Socially and economically disadvantaged groups suffer disproportionally from infectious diseases in Europe [15,16]. In the 1990s, during times of economic hardship, individuals in Central and Eastern Europe resorted to mushroom harvesting in wooded areas, and thereby increased their risk of tick-borne encephalitis [17]. The economic crisis in Kosovo in 1999–2000 resulted in the abandonment of food stores with the subsequent rise in rodent populations which led to the emergence of tularaemia [18]. The 2007 mortgage foreclosures in the Californian housing market resulted in many abandoned homes with swimming pools, increasing breeding habitats for mosquitoes, which was linked to an uncharacteristically early seasonal increase in West Nile Virus cases [19].

(3) Public health system includes surveillance and reporting, research and development, animal and food safety and health care. However, current surveillance systems might not be adequately equipped to cope with the arrival and dispersal of “tropical pathogens” commonly associated with warmer temperatures [10,20]. Contamination of blood products from donors infected with known, unexpected or unknown pathogens represents a significant threat to the blood supply and thus to public health. Current microbial blood-safety practices might not be adequate to cope with global environmental change [20]. Research and development of novel surveillance systems and of pathogen reduction technologies for the blood supply might reduce the risk from these emerging threats [10,20,21]. Access to healthcare is an important determinant for early treatment for VBD which can help interrupt an outbreak by removing an infected host from the transmission cycle; however, for viruses such as dengue virus, the patient is viremic before becoming ill thereby providing a source of mosquito infection. 

Eight plausible threat scenarios facing the European Union by 2020 were formulated, based on different drivers for infectious diseases from the ECDC foresight study described above [2]. These threat scenarios were selected based on the plausibility of the event, potential severity of the scenario in terms of burden of disease, and relevance of the scenario to multiple EU member states. They were primarily intended for prioritization of public health interventions and health policy decision-making. These plausible scenarios were used to develop tangible steps to mitigate the potential public health fallout from such infectious disease threats [2]. One plausible scenario included a VBD outbreak triggered by environmental/climate change, travel and tourism, global trade, and social inequality. The VBD scenario considered a threat from the introduction of new disease vectors, which creates new opportunities for disease transmission; expanded ability of vectors to transmit pathogens (e.g., by mutation); and a shift in the transmission range of diseases, hosts, and vectors due to socio-economic factors and climate change. 

This VBD scenario from the foresight study had some similarities with actual VBD outbreaks in Europe that occurred subsequently: The large dengue outbreak in Madeira in 2012 was sparked by the importation of viremic air traffic passengers in an environment where the vector *Aedes aegypti* had recently been introduced [22]. Environmental and climatic conditions contributed to the upsurge of WNF in Southeast Europe in 2010 [23]. Social inequality was a factor in the emergence of malaria in Greece in 2009 where migrant workers from endemic countries were part of the malaria parasite introduction and transmission cycle [24]. However, regardless of the accuracy of such foresight studies VBD continue to emerge and spread in the European Union. Traditional public health strategies might not suffice to cope with the public health challenges associated with global environmental change. Global connectivity and rapid environmental changes in land use and climate call for novel strategies of infectious disease control. ECDC has developed a pragmatic approach to tackle these emerging threats which are described below. 

## 2. The European Environment and Epidemiology (E3) Network

**Figure 1:**
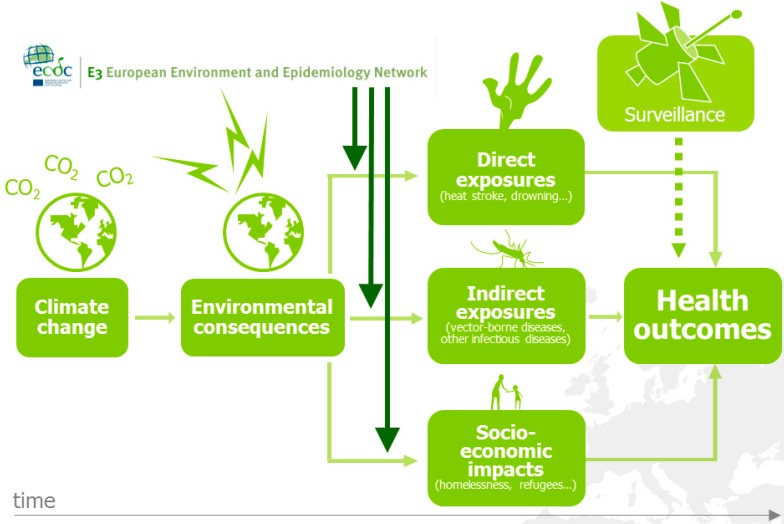
The European environment and epidemiology (E3) network.

Many drivers, like the ones identified in the foresight study above, can be considered epidemic precursors of disease (Figure 1). For example, monitoring changes in environmental and climatic conditions can help anticipate, or even forecast an upsurge of disease [10,13,21]. 

However, traditional environmental and infectious disease epidemiology is hampered by a number of shortcomings when it comes to the public health challenges from global environmental change [25]. Environmental, climatic, or epidemiologic data often lack historic baseline measurements which make comparison and extrapolation difficult. The effects of global environmental change do not adhere to typical effect-response relationships which traditional epidemiologic methods have been refined (and perfected) to measure. The pathways tend to be more complex and sometimes protracted; they can be direct but more often than not indirect, diffuse or delayed [26]. Estimating future health risks requires interdisciplinary collaborations to develop scenario-based models. Moreover, detection of health endpoints with traditional surveillance methods suffers from significant time lags due to delays in case identification, diagnosis, reporting, *etc.* which can result in exposure misclassification and confounding. A geographic shift in infectious diseases might also lead to an expansion of the disease burden into new areas and might therefore be missed. Thus, these changes in the risk profile for human populations call for novel approaches to assess interconnected and interdependent risks [12,27]. 

Rapid developments in Geographic Information Systems over the last decades have facilitated the management and use of spatial data for analytic epidemiology. A number of tools are now available over the web to explore and map spatial data that adhere to the standards of geographic data (e.g., INSPIRE directive: an infrastructure for spatial information in Europe to support Community environmental policies, and policies or activities which may have an impact on the environment) [28]. Risk models can then be used for the quantitative estimation of dynamic risks by taking into account changes in disease drivers. With this approach, future risk under different scenarios can be estimated. 

**Figure 2:**
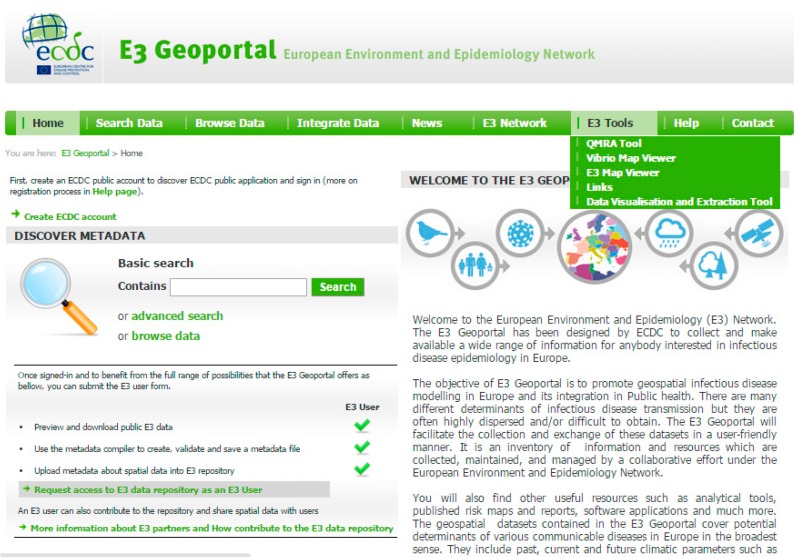
Screen shot of the E3 geoportal of the european environment and epidemiology (E3) Network [21].

ECDC has recognized the need for a proactive approach to deal with drivers of infectious diseases, identified in the foresight study discussed above [2,10,13,21]. ECDC has developed an infrastructure coined the European Environment and Epidemiology (E3) Network to help monitor environmental and climatic conditions related to infectious disease threats (Figure 1) [13,21]. The hub is composed of a data repository, a geoportal for data visualization, extraction and dissemination, and online tools that support the analysis of environmental, climatic and social drivers of infectious diseases (Figure 2) [29]. The E3 Network provides technical support for the reporting, monitoring, analysis, and mapping of data and enhances the analytical capacity of existing resources in Europe. Results have been disseminated to policy makers, public health practitioners, European Union and international agencies, other governmental sectors, and non-governmental organizations. 

**Figure 3:**
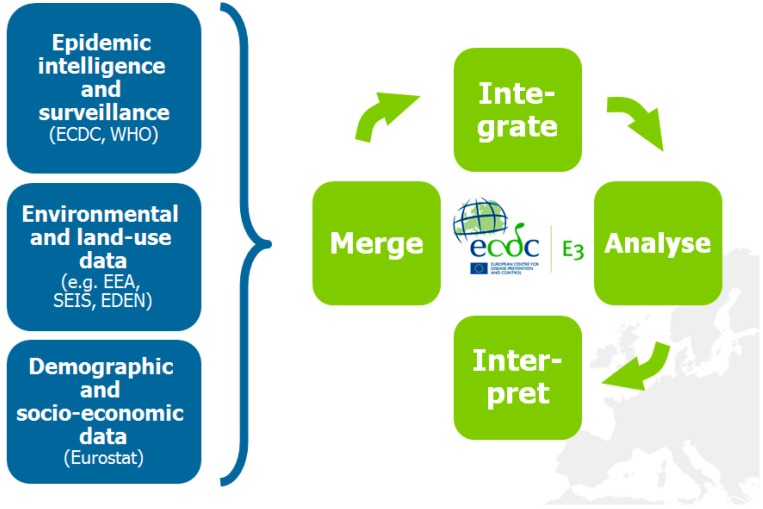
The concept of the European environment and epidemiology (E3) network.

With the E3 Network, climatic, weather environmental and other data can be merged and integrated with health data in order to provide support tools for decision makers (Figure 3) [10,13,21]. Easy and rapid linkage of data for novel analyses provides novel opportunities to deal with the complex nature of interconnected and interdependent risks. Such an approach can rapidly identify geographic areas of increased risk at a certain point in time. It can also define high risk populations that are particularly vulnerable to exposure and guide public health interventions. Information from these analyses can provide lead time for outreach to the public and engagement of health care providers. It can also be used to set public health policies and inform civil society about potential consequences of global environmental change on public health.

The initial building-block of the E3 Network was a set of data that was assembled through a research project of the Directorate-General for Research and Innovation of the European Commission entitled Emerging Diseases in a Changing European Environment project (EDEN). The processing of these data sets, and those continuously assembled from other sources, with regular outputs from advanced scientific analysis, serve as a reference point [29]. It also supports data exchanges and scientific collaborations between member states, researchers, ECDC experts and other authorised users across geographical and political boundaries in the European Community, with particular interest in the area of climatic change adaptation, landscape epidemiology and emerging disease threats. The data of the E3 Network have been used in a number of case studies, three of which are briefly described below. 

## 3. Intercepting Vector-borne Disease Emergence and Spread: Case Studies 

### 3.1. Environmental Suitability of Malaria Transmission in Greece

E3 data were used to predict the environmental suitability of malaria transmission in Greece [24]. In the past, malaria was endemic in Greece, but due to a successful malaria control and elimination programmes the country was declared malaria-free in 1974 [30,31]. Yet, importation of malaria has continued to occur accompanied by sporadic autochthonous transmission [32,33,34]. A cluster of six locally acquired *Plamosdium vivax* cases without travel history to an endemic area was discovered in 2009; a total of 267 malaria cases were noted by Greek health authorities between 2009 and 2012, although some reported a travel history [31]. Nevertheless, the continuing transmission of *P. vivax* by indigenous vectors in areas with permissive environmental and climatic conditions could potentially signal the re-emergence of malaria in Greece. Delineating specific areas environmentally suitable for transmission could direct and focus malaria control efforts. In order to assess the location of exposure of locally acquired malaria for such a suitability map, a standardized questionnaire was administered to each malaria case in Greece by a health officer. Cases with a travel history were excluded from this analysis as the goal was to delineate the areas environmentally suitable for autochthonous malaria transmission in Greece. By defining the environmental and climatic profile of areas with active transmission cycles between 2009 and 2012 other areas at risk for malaria re-emergence in Greece could then be defined. 

**Figure 4:**
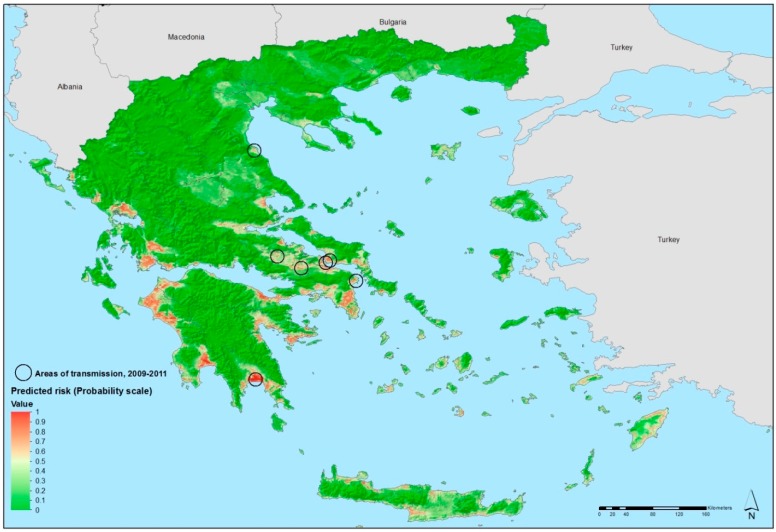
Areas latently hospitable and environmentally permissive for persistent malaria 16 transmission, Greece, 2009–2012. Map showing areas predicted to be environmentally 17 suitable for malaria transmission. Note: Values from 0 to 0.5 (dark to light green) indicate 18 conditions not favorable for malaria transmission (based on locally acquired cases); yellow 19 to dark red areas delineate conditions increasingly favorable for transmission (values from 20 0.5 to 1). Reprinted with permission from [25].

Geo-referenced environmental and climatic information for Greece and several other data sources were retrieved from the E3 Network data repository and other sources and processed for spatial modeling [35,36,37,38]. They included demographic indicators, land cover categories, altitude, seasonal variations of vegetation, temperature, *etc.* Using non-linear discriminant analysis (NLDA) available in eRiskMapper version 1.1.4 [39] a disease risk map was developed of areas suitable for persistent malaria transmission (Figure 4) [24]. 

Areas of environmental suitability for malaria transmission were characterized by warmer temperatures; low elevation; intensive, year round irrigated agriculture with complex cultivation patterns. Elevated temperatures (both nighttime and daytime temperature parameters were predictive in this model) can accelerate mosquito and parasite development which are likely contributing factors to mosquitoes presence and malaria transmission. This suitability map matched the historical distribution of malaria in Greece, particularly in the Peloponnese, the west coast of Central Greece and Epirus, and the east part of central Greece. 

This map was shared with public health practitioners in Greece responsible for integrated preparedness and response activities. Using EU structural funds, transmission was eventually interrupted in 2013 through targeted epidemiological and entomological surveillance, vector control activities, and awareness rising among the general population and health workers, in the areas environmentally suitable for transmission.

### 3.2. Environmental Determinants of West Nile Virus Transmission 

Transmission of West Nile virus (WNV) is determined by environmental/climatic and biological drivers [5,23]. In order for sustained transmission to take hold at a given place and time, susceptible birds have to come in contact with infected vectors. The avian transmission cycle is then amplified by local birds at which point the transmission can spill over to dead-end hosts such as humans or horses through bridge vectors that feed on both birds and humans/horses [23]. A crucial aspect of WNV amplification among competent insect vectors and vertebrate hosts is also their population densities which determine the intensity of transmission. Vector population densities depend on temperature which accelerates the growth rates [40]. Moreover, elevated temperature decreases the timing between blood meals but accelerates viral replication rates and thus the transmission of WNV [40]. Thus, permissive weather and environmental conditions are responsible for sustained local transmission whereas migratory birds and short distance vector transportation affect the geographic dispersal. 

**Figure 5:**
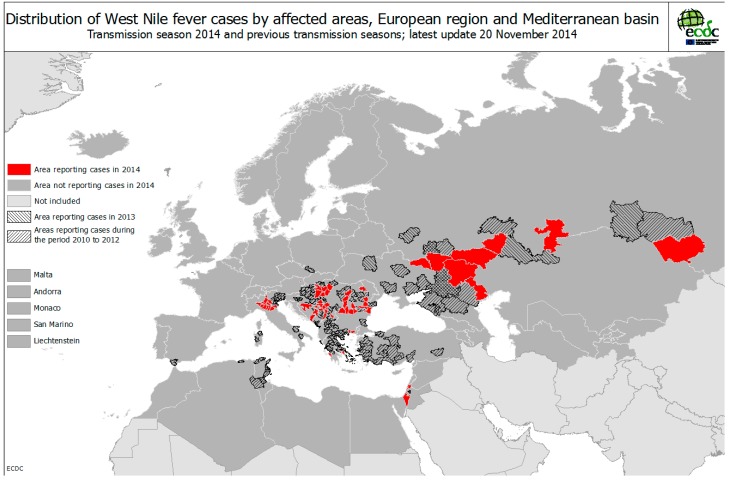
Distribution of WNF cases by affected areas, European region and Mediterranean basin, up to November 2014. Map reproduced with permission from ECDC 2014 **^©^** [48].

In Europe, several WNF outbreaks have been linked with elevated ambient temperature [41,42,43]. For example, Southeastern Europe was afflicted by a heat wave at the end of July to mid-August of 2010 which was followed by an outbreak of WNF cases [43]. Temperature deviations above the 30 year mean struck Russia (deviations > 9 °C), Romania (>5 °C), Turkey (>5 °C), and Greece (>3 °C) where 419, 57, 47, and 262 cases of WNF were reported, respectively (Figure 5). A number of meteorological variables were examined but temperature was the most significant one: in “colder” countries of more northern latitudes a statistically significant correlation between number of WNF cases and temperature was observed, with time lags of up to four weeks from the onset of the temperature raise; in contrast, “warmer” and more southern countries presented correlations without these time lags [43]. It has been noted that eruptions of WNF in previously unaffected areas tend to occur in years when summer temperatures deviate from the norm and that continued transmission can occur the following years even at normal summer temperatures [40]. The notion that the initial outbreak is associated with a heat wave but not the subsequent ones has been observed in a number of settings [40,43,44,45,46]. 

In order to examine other environmental variables as predictors of WNF risk [47] we tested the contribution of remotely sensed temperature, the state of vegetation and water bodies, and bird migratory routes. The analysis was performed at the district level where each district was considered “infected” if WNF human cases were reported there that year, and as “non-infected” otherwise.

**Figure 6:**
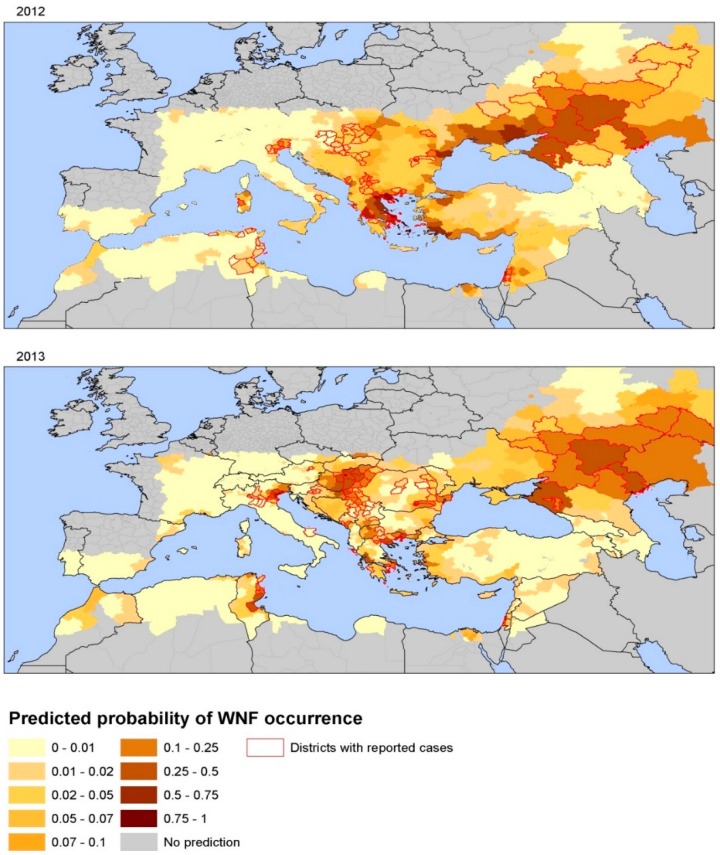
Map of predicted probability of WNV infection based on environmental predictors, Europe and neighboring countries, 2012 and 2013. Note: Reprinted with permission from [49].

The number of WNF cases from 2002 to 2011 was assembled from ECDC surveillance data, peer-reviewed papers and the grey literature to fit the models [49]. ECDC surveillance data for 2012 and 2013 were used for external validation. We used univariate and multivariate logistic regression models to assess the probability of a district to be categorized as WNV positive [49]. The status of infection was set as the response variable, and anomalies of temperature, wetlands, bird migratory routes, *etc*. as explanatory variables. In the final multivariate logistic regression model, parameters of WNV risk at district level for 2002–2011 were: July temperatures anomalies, the anomaly of the Modified Normalized Difference Water Index (MNDWI) [50] in early June, an outbreak the previous year, the size of the human population, wetlands and the type of avian flyways [49]. Model validation with 2012 and 2013 data showed a good level of prediction; thus, July temperatures anomalies and MNDWI can be considered determinants for WNV transmission in Europe. Summer temperature anomaly before the upsurge as the main driver of the outbreak was also confirmed in independent analyses, along with other ecological variables such as occurrence of irrigated croplands and highly fragmented forests [51]. These models indicate that risk maps for WNV transmission can be assembled with up-to-date anomalies of July temperatures for a given year along with the MNDWI [49]. These two environmental determinants lend themselves for an integration of environmental monitoring in public health surveillance systems of human cases, serological surveillance of domestic and wild avifauna, and entomological surveillance [47,52,53]. Figure 6 displays the spatial heterogeneity of the probability of WNV infection per district in 2012 and 2013 as predicted by this model [49]. Central and Eastern Europe, Turkey, Israel, and Tunisia were predicted to have higher risk values for 2012. In comparison with Figure 5, WNF cases were notified in all of the predicted high risk areas, excepted in Ukraine, and Turkey. Tunisia, Northern Italy, Northern Greece, Central Europe and South Russia scored the highest predicted values in agreement with main areas of transmission in 2013 (Figure 6). These findings indicate that the variables in this model can in part describe the variability in WNV transmission in Europe at the district level. Applying temperature anomalies for July can produce short-term and even long-term predictive maps of the probability of WNV infections. 

Needless to say, besides these environmental/climatic drivers, biological drivers such as the presence and abundance of avian hosts and mosquito vectors of WNV need to be better described in order to more accurately predict the transmission of WNF in Europe [23]. 

### 3.3. Dengue Dispersal through Air Traffic 

Dengue is by far the most significant mosquito-borne viral disease affecting humans globally but there is currently no efficacious vaccine available for dengue [54,55]. Tens of millions of cases occur annually resulting in approximately 20,000–25,000 deaths predominantly in children [54,56,57,58,59]. Transmission occurs largely in tropical and sub-tropical regions of the world, threatening almost half of the world’s population [58]. In continental Europe limited outbreaks have occurred in areas infested by two of the mosquito vectors, *Aedes albopictus* and *Aedes aegypti*. *Aedes aegypti* is the predominant mosquito vector that transmits the dengue virus to humans, whereas *Aedes albopictus* is a less effective vector [60]. *Ae. aegypti* is not present on continental Europe but, was first reported in 2005 on the Portuguese island of Madeira and has subsequently invaded the southern part of the island [61]. In Europe, *Ae. albopictus* has been reported in at least 15 countries (either established or recently recorded) and continues to broaden its reach. The development period for *Ae. albopictus* begins in April and dwindles off in October/November based on entomological monitoring activities in the Mediterranean [62,63,64]; however, the time of peak activity for *Ae. albopictus* are the summer months. 

Travelers from the tropics or subtropics, can be considered at risk for dengue virus (DENV) infection [65]. Through international air travel, infected travelers can arrive in Europe during their viremic period, and be bitten by local *Aedes* mosquitoes [66]. These infected mosquitos can subsequently transmit DENV locally and trigger an outbreak. In Europe, transmission has in fact occurred in areas where *Aedes* mosquitoes are present [67,68]. In 2010, two dengue cases without recent travel history or blood transfusion were recognized in Southern France [67] and two other dengue cases in Croatia [68]. Thus, for the first time in decades, local transmission has occurred in Europe. In 2012, an epidemic of over 2000 dengue cases erupted in Madeira, Portugal in areas where *Ae. aegypti* is known to be present [61]. 

**Figure 7:**
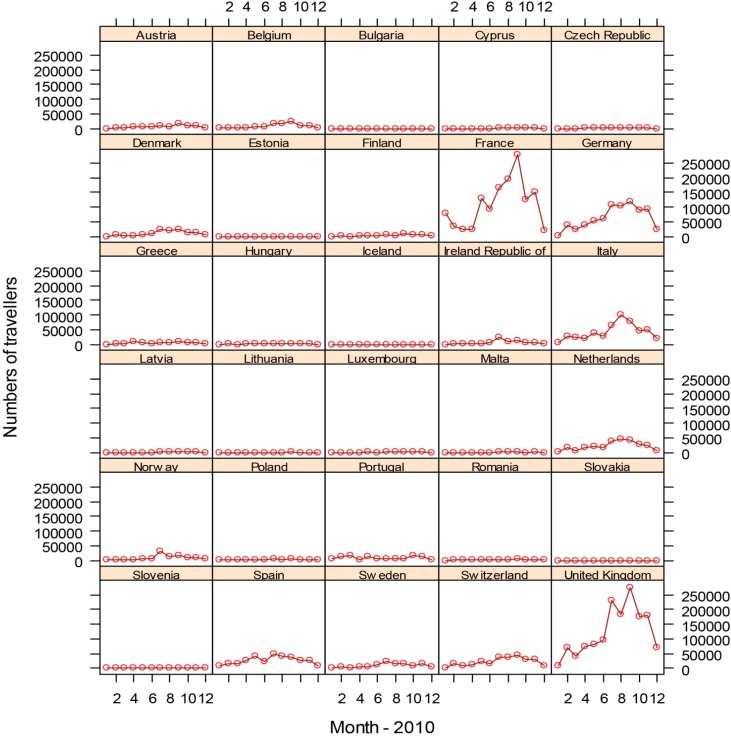
Country-level destination of international air travellers from dengue affected areas, by month 2010. Reprinted with permission from [3].

**Figure 8:**
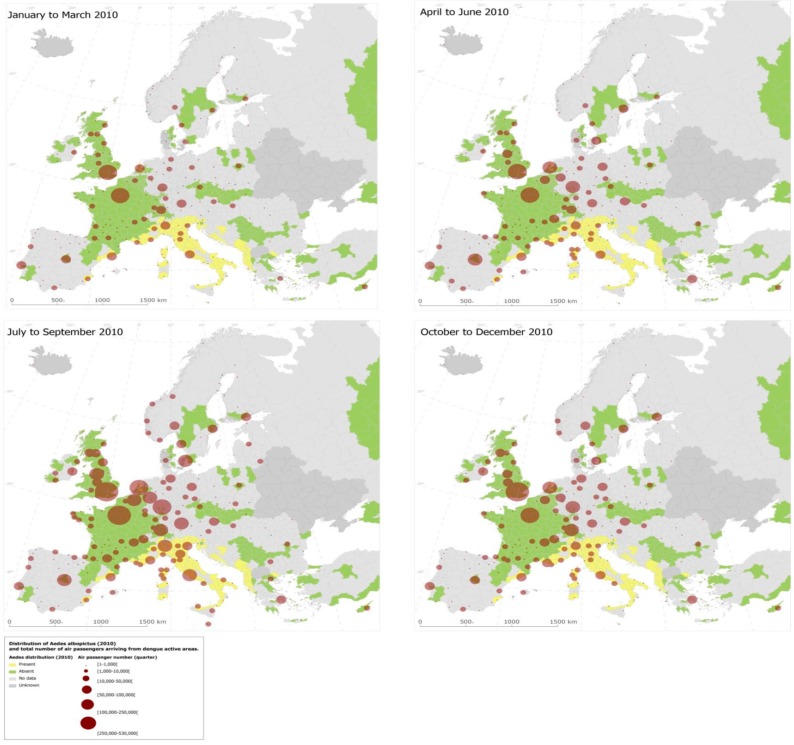
Airport-level final destination of international travellers from dengue affected areas. Reprinted with permission from [3].Disease and vector dispersal through international air traffic is the inevitable consequence of globalization. Pathogen introduction is difficult to intercept and public health has to resort to early detection, rapid response and effective control measures in order to contain potential dengue establishment and spread [53,69]. The approach presented here could be translated to other settings in support of integrated surveillance of human cases and vectors [3,10,70]. Such empirical models lend themselves to guide public health responses and can be developed into early warning systems of emerging risks [21,71].

With the goal to quantify the risk of dengue importation in areas where local transmission could occur (due to the presence of the vector) we took into account the global disease burden and seasonality of dengue, the volume and seasonal fluctuations of travelers originating from dengue-affected areas, and the seasonality and distribution of competent mosquito populations within Europe [3]. We developed a model based on 2010 data that relates air travelers from dengue affected areas to dengue importation to Europe. Over 5.8 million air passengers entered Europe from dengue-affected areas in 2010; country-level arrival by month is illustrated in Figure 7 [3]. The final European destinations was mapped as a function of the volumes of global air travelers arriving from areas with dengue activity during 2010; the spatial extent of the *Ae. albopictus* distribution (from the E3 data repository) was overlaid (Figure 8). Milan and Rome received over half, and Barcelona 14 % of these travelers that enter Europe from dengue-active/affected areas [3]. 

Imported dengue cases were significantly related to the monthly number of travelers arriving from dengue-affected locations. We developed a hierarchical multivariate model for imported dengue cases in 2010: the adjusted incidence rate ratio was 1.09 with a 95% confidence interval (95% CI) of (1.01–1.17) for every 10,000 traveller increase [3]. This corresponds to a 9% increase in the incidence of imported cases for every additional 10,000 travelers arriving from dengue-affected areas, all other predictors in the model being constant. In August, September, and October the rate ratio was 1.70 (95% CI: 1.23–2.35), 1.46 (95% CI: 1.02–2.1), and 1.35 (95% CI: 1.01–1.81), respectively [3]. 

This empirical model for 2010 aims to quantify the association between the number of monthly in-coming travelers with the number of monthly dengue importations at the country level. The main driver of dengue importation and its pattern into EU countries can be described with high spatial and temporal resolution international air traffic data (Figure 7) [3]. Moreover, the model accounts for dengue seasonality in the origin countries since dengue presence was recorded by month and documents that the importation risk for 2010 was the highest between August and October.

## 4. Conclusions

Vector-borne diseases are a threat to global public health, including Europe. Mounting an effective public health response to these threats obviously includes surveillance, awareness rising among the general public, public health practitioners, and policy makers about disease vectors and their relationship with infectious diseases. Exposure prevention through personal protection and vector control are essential activities of effective public health practice. However, by intercepting the emergence and spread of vector-borne diseases the human and financial costs of a potential epidemic can be contained [72]. Monitoring environmental/climatic precursors of these outbreaks through early warning systems can help predict the receptivity for introduction, emergence and spread of vector-borne diseases [21,71]. Forecasts and predictions can be developed by linking the monitoring of environmental/climatic precursors to dedicated disease surveillance systems with integrated vector surveillance of invasive and endemic vector species [53]. 

In recognition of the above, the European Commission emphasizes the need to strengthen public health preparedness, including surveillance and monitoring. Specifically, DG SANCO acknowledges the importance of the E3 Network: “By connecting these sources of information, the E3 network should bolster European early warning for climate-related disease events. It should also enable forecasting and risk mapping of infectious disease incidence in relation to environmental changes” [73]. Monitoring the upstream environmental/climatic and socioeconomic drivers of disease can provide the lead time for a swift public health response in order to contain human and financial costs associated with VBD emergence and spread in the European Union. 
